# Achieving fruit, juice, and vegetable recipe preparation goals influences consumption by 4th grade students

**DOI:** 10.1186/1479-5868-4-28

**Published:** 2007-06-29

**Authors:** Karen W Cullen, Kathy B Watson, Issa Zakeri, Tom Baranowski, Janice H Baranowski

**Affiliations:** 1 Department of Pediatrics-Children's Nutrition Research Center, Baylor College of Medicine, 1100 Bates St., Houston, TX 77030, USA

## Abstract

**Background:**

Including children in food preparation activities has long been recommended as a method to encourage children's consumption, but has not been evaluated. Goal setting is also a common component of behavior change programs. This study assessed the impact of attaining goals to prepare fruit-juice or vegetable recipes on student fruit and vegetable consumption as part of a 10-week fruit and vegetable intervention for fourth grade students.

**Methods:**

At six of the 10 sessions, students (n = 671) selected a fruit-juice or vegetable recipe to prepare at home before the next session. Students returned parent-signed notes reporting their child's goal attainment. Baseline and post consumption were assessed with up to four days of dietary recalls. Analyses included regression models predicting post consumption from the number of fruit-juice or vegetable recipe preparation goals attained, controlling for baseline consumption.

**Results:**

In general, girls and Hispanic students achieved the most recipe preparation goals. For students with highest baseline fruit-juice consumption, post fruit-juice consumption was higher by about 1.0 serving for those achieving 2 or 3 fruit-juice recipe preparation goals. Post vegetable consumption was highest for students reporting the highest baseline vegetable consumption and who achieved two or three vegetable recipe preparation goals. In general, recipe goal setting was a useful procedure primarily for those with high baseline consumption.

**Conclusion:**

This is one of the first reports demonstrating that home recipe preparation was correlated with dietary change among children.

## Background

Children's participation in food preparation activities has long been advocated as a method to increase consumption [1]. Several studies have demonstrated positive outcomes from food preparation activities. For example, cooking classes or food sampling increased preparation of the foods by adults with Type 2 diabetes [2]; improved attitudes toward eating healthy by low-income women [3]; and increased cooking skills and attitudes among college students [4]. A program of 10 cooking sessions in the classroom for kindergarten through 6^th ^grade students resulted in increased preferences and knowledge, and decreased plate waste [5]. Parents have reported their belief that children's participation in food preparation activities would increase child preferences for the foods [6].

Goal setting is both an important skill in self-controlling behavior [7], and a frequently used procedure to change behavior [8,9]. While many nutrition education programs employ goal setting as a dietary change procedure, there has been little published evidence relating goal setting activities, including goal achievement, to children's dietary behavior change [8]. In one study, complex relationships were detected: students with low baseline fruit-juice preferences who achieved more fruit-juice goals increased post fruit-juice consumption more; those with low baseline vegetable consumption who achieved one vegetable goal consumed more vegetables; and for boys and those with high baseline fruit, juice, and vegetable consumption, achieving three general goals was related to increased fruit, juice, and vegetable intake [10]. Thus, goal setting with goal achievement appears to be a useful dietary change procedure for students. Little is known, however, about goal setting for behaviors associated with consumption, e.g. setting goals to prepare recipes.

Setting goals to prepare fruit, juice, and vegetable recipes was an integral component of the *Squire's Quest! *interactive multimedia computer program for 4^th ^grade children [11]. This study assessed the relationship of goal achievement for fruit and vegetable recipe preparation to increases in fruit and 100% fruit juice or vegetable consumption. Contextual factors (e.g. baseline fruit and juice or vegetable preferences, self-efficacy, and consumption) that could influence recipe goal setting and attainment also were assessed [12,13].

## Methods

### Study Sample

Approximately 70% of eligible fourth grade students (n = 1578) from 26 Houston area elementary schools agreed to participate in the evaluation of the *Squire's Quest! *program and were randomly assigned to treatment or control conditions. *Squire's Quest! *was approved by the Institutional Review Boards of the University of Texas M.D. Anderson Cancer Center and Baylor College of Medicine. Students and guardians signed informed assent and consent, and guardians completed a demographic form. Students who could not understand English were excluded since budget limits precluded other language versions of the program. Only students in the 13 schools randomized to the treatment condition were included in this analysis, since control group students did not use *Squire's Quest *or set goals.

### Intervention: *Squire's Quest!*

*Squire's Quest! *is an adaptation of the successful *Gimme 5 *classroom curriculum to increase fruit, juice, and vegetable consumption to a 10-session interactive multimedia formatted game [11,14]. *Squire's Quest! *is based on social cognitive theory (SCT) [7] and the content includes activities promoting increased asking behavior, food preparation, produce shopping, fast food selection, problem solving, goal setting, self-regulation, and self-reward skills in regard to eating fruit, juice, and vegetables. All the sessions included setting a goal to eat a fruit or vegetable at a specific meal or place [10]. In six of the sessions, students selected a goal to prepare a fruit-juice or vegetable recipe from a menu of recipes, and prepared it in the virtual kitchen. The student was guided to prepare the recipe at home and a copy printed out for home use. There were six fruit-juice and nine vegetable recipes (Table 1). Each student was assigned a separate FV dietary change goal, and then chose a recipe preparation goal at each of the six sessions that included recipe preparation. A parent or guardian signed a form verifying whether each goal was achieved by the child. Signed sheets were returned to the school by the student and entered into the program database by a project dietitian. The software provided positive rewards for attained goals (e.g., points to obtain levels of knighthood), and encouraged the participant to problem solve or otherwise continue toward unattained goals [11].

**Table 1:** *Squire's Quest! *Recipes and number of students who selected and completed each

		Students completed	
		
Session	Recipe	n	(%)
1	Razzle Dazzle (fruit juice mix)	453	68
2	Royal Slush (fruit slushy)	335	50
	Milky Way (cinnamon juice beverage)	0	0
4	Veggie Power (Mexican corn and lima beans)	0	0
	Chef Karat's Special (glazed carrots)	125	19
	Jester's Sweet Potato Surprise (sweet potato and pineapple mix)	67	10
5	Round Table Pizza (vegetable pizza)	80	12
	French Fry Fantastic (seasoned oven fries)	92	14
	Wizard's Magic Pocket (pita pocket/veggie sandwich)	228	34
8	Golden Knight Burrito (veggie burrito)	200	30
	Heart-y Rice (Rice and vegetable dish)	107	16
	Stone Soup (vegetable soup)	74	11
9	Celebration Sundae (fruit parfait)	82	12
	Fruit and Chocolate in the Round (Fruit and dip)	38	6
	Great Shake (Fruit smoothie treat)	214	32

### Study design

Students completed four days of dietary assessment during a two week baseline period prior to beginning the program. Each child completed two sessions of *Squire's Quest! *per week for five weeks, and then four days of post dietary assessment during the next two weeks. The students included in these analyses were restricted to those children (n = 671, 84%) who completed 9 or 10 sessions, and thereby had the opportunity to achieve the majority of available goals.

### Instruments

Dietary intake data were collected at baseline and post intervention for 4 days using the Food Intake Recording Software System (FIRSSt) [15]. FIRSSt is a computerized 24-hour dietary recall program designed for use by fourth grade students that emphasizes the assessment of fruit, 100% juice, and vegetable consumption. Servings of fruit and 100% fruit juice were summed, as consumption of juice was low. This method of dietary data collection was validated [15]. Trained data collectors initially instructed children in the use of the FIRSSt food recording system and the *Squire's Quest! *computer game.

Preferences were measured for 17 vegetable, 10 fruit and three 100% fruit juice items [12,13]. Students were asked to select how much they liked each item on a 3-point scale: 0 = not like, 1 = like a little, and 2 = like a lot. Responses were summed to form separate fruit-juice and vegetable preferences scales. These scales had acceptable internal consistency reliability (α = 0.82 for fruit-juice; 0.80 for vegetable) [12,13].

Self-efficacy for eating fruit, 100% fruit juice, and vegetables was measured by three scales: fruit, 100% fruit juice, and vegetable substitution self-efficacy (8 items, alpha = 0.87); fruit, 100% fruit juice, and vegetable availability self-efficacy (10 items, alpha = 0.80); and eating fruit, 100% fruit juice, and vegetable self-efficacy (5 items, alpha = 0.72). The questions were rated on a 5-point scale from agree a lot to disagree a lot, with higher scores representing higher self-efficacy [13,16].

Parents self-reported their level of education. Percent of students receiving free or reduced price lunches was used as a surrogate measure of socioeconomic status (SES). Schools with less than 40% of their students receiving free or reduced lunch meals were categorized as middle income, while schools with 40% of their students or higher receiving free or reduced lunch meals were categorized as low income. The school district had a large number of low-income students so this cutoff was selected to achieve two SES categories.

### Analysis

Percent of fruit-juice and vegetable recipe preparation goals achieved were calculated and compared across gender, ethnicity, mother's education and SES categories. To evaluate the impact of goal achievement on consumption, analyses included regression models using hierarchical backward elimination procedures predicting post fruit-juice or vegetable consumption from the numbers of fruit-juice and vegetable recipe preparation goals achieved, respectively. For all models, baseline values for fruit-juice and vegetable intake, fruit-juice and vegetable preferences, the three self-efficacy scales, and change in preferences and self-efficacy from baseline to post-assessment were added to the model, in addition to controlling for gender, ethnicity, high or low percent free/reduced school lunch, and mother's education. Since several variables were demonstrated to moderate goal attainment and consumption [10], and no one has previously studied this phenomenon, all 2-way interaction terms were also included in each model and backward deleted. During each analysis, students with missing data were not included. Significance level was <0.05. Data were analyzed using the Statistical Package for Social Sciences (SPSS version 10.1 for Windows, 2000, SPSS Inc., Chicago).

## Results

### Sample characteristics

Thirteen schools were randomly assigned to the intervention group of *Squire's Quest! *(48% males, 18% white, 43% African-American, 31% Hispanic, and 8% Asian/other) (Table 2). The majority of the 4^th ^grade students were Hispanic or African-American, reflecting the demographics of the school district, and low income, attending schools with greater than 40% of the students receiving free or reduced price lunches. Mean age or the students was *9.4 (± 0.6) years*. Students who completed 9 or 10 sessions (n = 644 vs. 116) were more likely to be Asian/Other or White (p = 0.012); have a mother who had a bachelor's degree or higher (p = 0.003); and to attend higher SES schools (p = 0.000) (Table 2). No significant difference was found by gender.

**Table 2:** Characteristics of fourth grade students enrolled in the *Squire's Quest *nutrition education program by number of sessions completed.

Characteristic	Total n (%) ^a^	Completed 9 or 10 sessions n (%)	Completed < 9 sessions n (%)
Gender Males	369 (47.2)	319 (48)	50 (43.1)
Females	412 (52.8)	346 (52)	66 (56.9)
Ethnicity* White	125 (16.5)	116 (17.9)	9 (8.4)
African American	339 (44.8)	279 (43.0)	60 (56.1)
Hispanic	234 (31.0)	200 (30.8)	34 (31.8)
Asian/Other	58 (7.7)	54 (8.3)	4 (3.7)
Mother's Education** High school graduate or less	303 (42.3)	252 (40.9)	51 (50.5)
Some college/business school/vocational school	204 (28.5)	169 (27.4)	35 (34.7)
Bachelor's degree or higher	210 (29.3)	195 (31.7)	15 (14.9)
School Free/Reduced Lunch*** Low socioeconomic status (>50% of students eligible for free or reduced price meals)	546 (68.8)	441 (65.7)	105 (85.4)
Higher socioeconomic status (<50% of students eligible for free or reduced price meals)	248 (31.2)	230 (34.3)	18 (14.6)

*Chi-square test, p ≤ .05

**Chi-square test, p ≤ .01

*** Chi-square test, p ≤ .001

^a^Numbers do not add up to the total sample size (n = 671) because some response data is missing due to self-reported questionnaires

Daily consumption at baseline was 1.6 servings fruit, 0.8 serving 100% fruit juice, and 0.8 serving non fried vegetables. An average increase of 1.0 fruit, 100% fruit juice, and vegetable servings, combined, was achieved at post assessment [11].

### Children's Recipe Goal Setting

In general, girls achieved more recipe preparation goals than boys (Table 3). For fruit-juice recipe goals, Hispanic students achieved more than white or African-American students; white students achieved more than African-American and Asian/other students; and Asian/other students achieved more than African-American students. For vegetable recipe preparation goals, Hispanic students attained more than white or African-American students. Students whose mothers achieved a high school education or less achieved more fruit-juice recipe goals than students whose mothers reported some college or higher educational achievement. There were no differences in recipe preparation goal achievement by school SES (Table 3).

**Table 3:** Differences in fruit-juice and vegetable recipe goal attainment by gender, ethnicity, mother's education and school socioeconomic status

		Percent Fruit-Juice Recipe Goals Attained		Percent Vegetable Recipe Goals Attained	
	
	n	Mean	Std. Dev	Mean	Std. Dev
Gender					
Boy	319	56**	36	53***	41
Girl	346	65	35	65	37

Ethnicity					
African-American	279	53*** ^a^	35	49*** ^ad^	39
Asian/other	54	64^bd^	33	65^bd^	37
Hispanic	200	72^bc^	32	74^bc^	35
White	116	61^d^	37	57^d^	40

Parent education					
High school graduate or less	252	68** ^a^	34	65	39
Some college/business school/vocational school	169	58^bc^	35	55	39
Bachelor's degree or higher	195	56^bc^	37	57	38

School Socioeconomic Status					
low income (>50% of students eligible for free or reduced price meals)	441	62	34	61	39
high income (<50% of students eligible for free or reduced price meals)	230	58	37	56	40

* p < 0.05; **p < 0.01; ***p < 0.001.

Within subgroups and columns, numbers with the different superscripts are significantly different.

Baseline fruit-juice consumption was a significant bivariate predictor of post fruit-juice consumption (p < 0.001), thereby making other variables in the model predictors of change in fruit-juice consumption from baseline. An interaction term, baseline fruit-juice consumption and attaining 2 or 3 fruit-juice recipe preparation goals, was significantly related to post fruit-juice consumption (p < 0.01) (Table 4). Other significant predictors of post fruit-juice consumption not included in the interaction term were achieving one fruit-juice recipe preparation goal (p < 0.05), higher baseline fruit preference (p < 0.01), and mothers with high school or less education (compared with some college) (p < 0.01) (r^2 ^= 0.21). For students with highest baseline fruit-juice consumption, post fruit-juice consumption was higher by about 1.0 serving for those achieving 2 or 3 fruit-juice recipe preparation goals. Post fruit-juice consumption did not appreciably vary by number of recipe preparation goals achieved for children with mean or lower baseline fruit-juice consumption.

**Table 4:** Results from Stepwise Linear Regression (Backward Elimination) of the Natural Log of Post Fruit and Juice Consumption (plus 1) Controlling for Baseline Fruit and Juice Consumption

Variables	Unstandardized Coefficients	Std. Error	Std. Coefficients	t	Sig.
(Constant)	0.33	0.21		1.58	0.115
Fruit and Juice Baseline Consumption	0.07	0.01	0.26	4.83	0.000
Fruit Preference	0.02	0.01	0.11	2.76	0.006
1 FJ Recipe Goal Met	0.18	0.09	0.11	1.97	0.050
2 or 3 FJ Goals Met	-0.05	0.09	-0.03	-0.50	0.615
Mother's Education (Referent:High School or Less)					
Some college/business school/vocational school	-0.20	0.07	-0.13	-3.09	0.002
Bachelor's degree or higher	-0.10	0.06	-0.07	-1.62	0.105
Baseline Consumption by 2 or 3 Goal Attainment Interaction	0.07	0.02	0.21	3.36	0.001
R^2 ^= .21					

Baseline vegetable consumption was a significant bivariate predictor of post fruit-juice consumption (p < 0.001), thereby making other variables in the model predictors of change in vegetable consumption from baseline. The interaction term of baseline vegetable consumption by achieving one vegetable goal was significantly related to post vegetable consumption (p < 0.05) (Table 5). Other significant predictors of post vegetable consumption (r^2 ^= 0.19) were higher baseline vegetable preferences (p < 0.001), gender (girl) (p < 0.05), and mothers with a college education or more (compared with high school graduate) (p < 0.001). Post vegetable consumption was highest for children who reported the highest baseline vegetable consumption and achieved two or three vegetable recipe preparation goals. Achieving a second or third goal did not improve vegetable consumption for those with mean baseline vegetable consumption, and post consumption declined for those with zero baseline consumption who achieved two or three goals.

**Table 5:** Results from Stepwise Linear Regression (Backward Elimination) of the Natural Log of Post Vegetable Consumption (plus 1) Controlling for Baseline Vegetable Consumption

Variables	Unstandardized Coefficients	Std. Error	Standardized Coefficients	t	Sig.
(Constant)	0.01	0.13		0.04	0.966
Vegetable Baseline Consumption	0.19	0.02	0.40	8.18	0.000
Vegetable Preference	0.01	0.00	0.11	2.81	0.005
<= 1 Vegetable Recipe Goal Met	0.11	0.05	0.10	2.06	0.040
Gender (Male = 1)	-0.09	0.04	-0.09	-2.17	0.031
Mother's Education (Referent:High School or Less)					
Some college/business school/vocational school	0.09	0.05	0.08	1.83	0.067
Bachelor's degree or higher	0.17	0.05	0.15	3.57	0.000
Baseline Vegetable Consumption by <= 1 Recipe Goal Met	-0.09	0.04	-0.12	-2.25	0.025
R^2 ^= .19					

## Discussion

The *Squire's Quest! *intervention was successful, resulting in a 1.0 serving increase in total fruit, juice, and vegetable intake [11]. The current analyses reveal that achieving fruit-juice and vegetable recipe preparation goals was related to post fruit and juice or vegetable consumption, but the magnitude and direction of the relationship depended on number of goals achieved and baseline consumption. Students with high baseline fruit-juice consumption (M+SD) who attained 2 or 3 fruit-juice recipe preparation goals reported highest post fruit-juice consumption compared with students with baseline fruit-juice consumption at the mean or lower. Students at mean baseline consumption had a slight benefit for achieving one recipe preparation goal, but none from achieving two or three goals. Those with zero baseline fruit-juice consumption had only modest benefit from one goal and no benefit beyond achieving one goal. Thus, recipe goal setting was a useful procedure primarily for those with high baseline consumption. These results are contrary to the intuitively expected linear increase in consumption with more goals achieved, but are consistent with the complex interactions obtained from the analysis of *Squire's Quest! *consumption goal setting [10]. Perhaps students who were consuming more fruit-juice at baseline were more interested in preparing fruit-juice recipes, more amenable to the goal setting process, and thereby benefited the most. Because food preparation occurred at home, the lack of support for such activities in some families might have been a barrier for some students. Perhaps some of the recipes could be prepared in the classroom, giving all students an opportunity to practice and become more interested in preparing and consuming the foods [5].

A significant increase in post vegetable consumption with the achievement of zero or one vegetable recipe preparation goal was detected for those with high baseline vegetable consumption. There was little additional consumption for achieving more preparation goals. The ideal number of goals for an intervention period is not known, and may differ based on preferences, consumption, and family factors. Only 30% of students reported completing session 4 goals which were all vegetable recipes. In contrast, 50–68% of the fruit recipe preparation goals were achieved. Session 8 recipes were vegetables, but included vegetable burritos, a rice-vegetable mixture and vegetable soup; 50% of these were achieved. Perhaps increasing vegetable consumption is more difficult than increasing fruit-juice consumption because of generally low preferences for and consumption of vegetables [12]. Perhaps the process of change takes more time than allowed in the sequencing of and time allotted in the game. Children might need several weeks to practice vegetable recipe preparation and change meal eating habits. This is an important area for future research.

Girls reported more success with recipe preparation goal setting than boys, which may be supported by findings that women were marginally more likely to be influenced by food sampling [2]. These differences in the effectiveness of recipe preparation goal achievement suggest this process may need to be tailored to specific baseline characteristics such as gender, preferences or consumption.

Better results for students whose parents had lower educational achievement were found for fruit-juice recipe preparation, while higher education of the parent was associated with more vegetable recipe preparation. Home availability of lower preferred vegetables may be a problem for lower income children. These results indicate the need for further research on gender and family educational differences in recipe preparation goal setting that may influence future application of goal setting in interventions.

There are several limitations that should be noted. Participants were from one large urban school district in southeast Texas which limits generalizability to fourth grade students in other areas or states, or to students in other grades. Although recipe goal achievement was verified by parental report, some parental verifications may not have been valid (which suggests that goal setting might be an even stronger predictor of change if we had more valid goal achievement data). No data were available on student access to ingredients or equipment in the home, or on parent experiences with student recipe preparation. Future research in this area should assess these important factors. There are also not data on dose, i.e. whether the attainment of two or three goals would have improved consumption. All data were collected by self-report which is subject to human error and minimizes the likelihood of detecting relationships [9]. To minimize the possible problem of interviewer induced error, all data were collected directly by computer and trained data collectors were present in all classrooms during the intervention to promote accuracy while students were completing their computer collected data. The dietary recall methods used in this study were validated, but were somewhat less accurate than dietitian-completed 24-hour diet records [15]. These results may be the result of chance by introducing interaction terms, but most of the tests were highly statistically significant. While capitalizing on chance is a possibility that needs to be cross-validated in future tests of these relationships, the findings were detected despite unreliability in all the measures.

These findings suggest that the true relationships between goal setting and achievement for recipe preparation and dietary intake are more complex than previously believed, and deserve more careful exploration. Consumption was measured before and after the 10 sessions, not when the student was to prepare the specific fruit-juice or vegetable recipes. Perhaps some goals were successfully achieved, but the change was not permanent, or not implemented at a level sufficient to be reflected in total consumption. These findings need to be replicated. No other data are available to which to compare the number of fruit-juice and vegetable recipe preparation goals achieved for change in fruit, 100% juice, and vegetable consumption.

The strengths of this study include a large sample of children in one grade (which limits the influence of developmental differences), a substantial dietary change (1.0 serving) resulting from the intervention, and multiple days of dietary assessment to enhance reliability of the dependent variable. Further intervention research should develop and test methods to increase the levels of child fruit-juice and vegetable recipe goal achievement; develop and test methods to reduce error in assessing recipe goal achievement (e.g., phone calls to parents); explain why some goals were related to dietary behavior change and others were not; identify the optimum number of goals per intervention or per unit of time; and assess whether gender and SES differences in these relationships might require tailoring of goals.

Although goal setting and food preparation are believed to be important components of successful dietary behavior change, little research has assessed whether goal setting contributes to successful intervention outcomes. In the current research with fourth grade children, modest effects were found for fruit-juice and vegetable recipe goal achievement in regard to dietary fruit, 100% fruit juice, and vegetable behavior change. Practitioners should be encouraged to include recipe goal setting in interventions with children, and include assessment of goal setting success in evaluations.

## Competing interests

The author(s) declare that they have no competing interests.

## Authors' contributions

TB, JB, and KC conducted the study. KW and IZ conducted the statistical analyses. KC wrote the manuscript, all authors edited the manuscript.
